# Resuscitation of neonatal critical care: a call for identity and course correction

**DOI:** 10.1038/s41372-026-02774-y

**Published:** 2026-06-30

**Authors:** Satyan Lakshminrusimha, Clara H Song, Robin H. Steinhorn

**Affiliations:** 1 Department of Pediatrics, University of California at Davis, Sacramento, CA USA; 2 Southern California Kaiser Permanente, Orange County, CA USA; 3 Department of Pediatrics, University of California San Diego, La Jolla, CA USA

**Keywords:** Health services, Health occupations

## Abstract

The field of neonatology is currently navigating a multi-faceted crisis involving professional identity, workforce sustainability, and financial equity. While the Neonatal Intensive Care Unit (NICU) serves as a vital economic engine for modern health systems and academic departments, the individual neonatologist faces increasing clinical acuity, documentation burdens, long work hours, and a widening compensation gap compared to adult counterparts. This manuscript explores the “Neonatology Paradox,” as a specialty with the highest productivity in a financially challenged department of pediatrics. We also evaluate the limitations of current productivity benchmarks and propose structural changes, including staffing model changes to optimize work hours, reduce productivity expectations, and work life integration. If these measures fail, we explore the potential for an independent department of Neonatal Critical Care to ensure the future of the workforce.

## Introduction: the changing landscape of neonatology

Neonatal care has undergone a radical transformation since the 1980s. Dr. Joseph Butterfield’s vision of “Newborn Country, USA” established a regionalized concept of care based on partnerships between university centers and community pediatricians [1]. The academic neonatologist in the 1980s was supported by a large resident and fellow workforce. The attending physician could round on their patients and take a break from clinical activities to pursue teaching or research while being on service. The attending physician had time to regularly call the referring pediatrician and provide feedback on every referral throughout the hospital stay so that there would be continuity of care after this patient’s discharge. Long stretches of service (4–6 weeks) associated with night calls from home were feasible. There were many procedures in the NICU—multiple intubations, chest tube placements, and exchange transfusions. The “tube thrill” and procedural excitement of the NICU attracted residents and enticed them to pursue a career in Neonatology. Extremely preterm infants and infants with complex congenital anomalies had relatively high mortality resulting in shorter lengths of stay.

## The current landscape: loss of “tube thrill” and the 6 Cs in modern neonatal care

The duration of time spent by residents in the NICU has decreased over the years. With the recent revision of the pediatric resident curriculum by the Accreditation Council for Graduate Medical Education (ACGME), residents often spend as little as 4–6 weeks in the NICU during their three-year residency [2]. As a result, new fellows in neonatal perinatal medicine require more support from the attending physicians. This has resulted in more attending physicians staying in house in the neonatal and pediatric ICUs. According to an Association of Administrators in Academic Pediatrics (AAAP) and Association of Medical School Pediatric Department Chairs (AMSPDC) survey conducted in 2024 and published in 2026, attending physicians are staying in 64% of NICUs, 71% of PICUs and 77% of CICUs in academic pediatric departments [3].

**Fig. 1:**
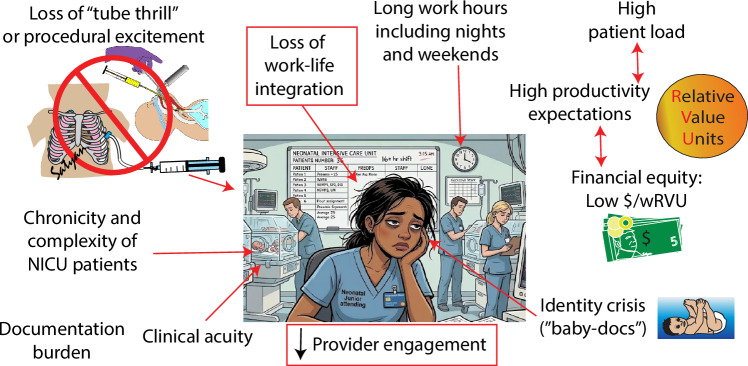
Current crisis in neonatal workforce resulting low physician engagement and loss of work-life integration. Created by Dr. Satyan Lakshminrusimha with some images created by Google Gemini.

With advances in neonatal critical care, survival of extremely preterm infants and infants with complex congenital anomalies has increased. With the advent of continuous positive airway pressure (CPAP), surfactants, inhaled nitric oxide, Rh immunoglobulin, and better surgical techniques, there are fewer procedures performed in the NICU. In some NICUs, critically sick infants such as those on extracorporeal life support (ECLS) and postoperative cardiac surgery are transferred to the PICU, further reducing procedures in the NICU. The modern neonatologist operates in a vastly different landscape defined by the “6 Cs”: increased **Clinical Acuity,** **Complexity,** **Chronicity,** **Consultative** nature of the work, and **Charting** requirements, all while facing a decrease in **Continuity** of care (Fig. 1).

## Identity crisis, chronicity and neonatal “Geriatricians”

The evolution of neonatal care has created a paradox: while “tiny babies” in NICUs are a substantial source of inpatient and total revenue to Children’s hospital systems and academic departments of Pediatrics [4], the neonatologists providing this care are experiencing a decline in engagement and professional euphoria replaced by exhaustion and a growing identity crisis [5]. The field of neonatology in the 1980s provided acute critical care to newly born and young neonates ( ≤ 28 days of postnatal age) with exciting innovations such as surfactant, inhaled nitric oxide and high-frequency ventilation. In the current landscape, providers feel like “neonatal geriatricians” providing chronic care to “older” infants with bronchopulmonary dysplasia (BPD), short-gut syndrome and other long-term sequalae of surgical conditions.

At the same time, closure of pediatric beds in community hospitals, and increased emergence of level II and level III NICUs, have required children’s hospitals to accommodate more non-neonatal pediatric patients and a high Medicaid load, adding to their financial burden. To address this financial burden, there is often an increased reliance of neonatology revenue to sustain the organization.

## The business of benchmarks and the cFTE denominator

The current model for physician compensation and productivity relies heavily on the work Relative Value Unit (wRVU). There is a prevailing myth that productivity is directly correlated to pay. However, data from 2024 reveals a stark discrepancy: while the median three-year wRVU productivity for an academic neonatologist (10,664) is nearly twice that of an academic emergency medicine or pediatric or adult critical care provider, yet the three-year median total compensation for a neonatologist is similar to pediatric critical care or emergency medicine and lower than adult pulmonary critical care. This creates the “Neonatology paradox”. Neonatology is typically the highest wRVU and revenue generating division within academic departments of pediatrics and yet the annual compensation is similar to fields that have nearly half the productivity (e.g., pediatric cardiology, critical care medicine) or considerably lower work hours (e.g., pediatric emergency medicine).

**Fig. 2:**
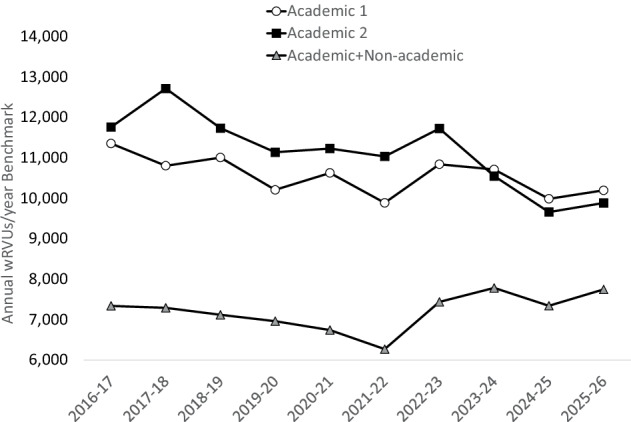
Productivity (wRVUs/1.0 cFTE) benchmarks from 3 sources in neonatal critical care. The first two are academic benchmarks. The third one is a combined academic/non-academic benchmark. The exact identity of the source of benchmarks is concealed due to the proprietary nature of the data.

Academic neonatology productivity benchmarks have decreased by 14% over the last decade. Health system leaders often misinterpret this decline. It does not reflect a decrease in work, but rather the failure of current CPT codes to capture the additional time required to provide in-house nocturnal attending coverage. In-house coverage by attending physicians has a paradoxical effect of reduced critical care billing through enhanced care (i.e., preventing decompensation or accelerating weaning) [6]. The lower level of experience among early phase pediatric trainees (residents and first year fellows) is secondary to decreases in NICU rotations due to ACGME pediatric curriculum changes and work hour restrictions. These changes necessitate increasing attending physicians’ presence for patient safety, reducing wRVU productivity per FTE. This gradual reduction in wRVU benchmarks for academic neonatal perinatal medicine was also replicated by benchmarks that extract combined data from academic and non-academic sources (Fig. 2).

Productivity benchmarks in neonatology are higher in academic settings compared to non-academic settings. These benchmarks are inherently unfavorable to academic physicians. Most academic physicians are given 10–20% protected time for educational and scholarly activities yet expected to generate similar wRVU relative to their non-academic counterparts [7, 8]. In addition, other important roles such as residency and fellowship program director, medical director, division head, and vice chair [9] reduce the clinical component of the full-time equivalent (cFTE) [8, 10]. Some benchmark surveys collect data on total wRVUs generated during the measurement period and divide by the cFTE to generate annual productivity/1.0 cFTE. However, the definition of cFTE varies between surveys, hospitals, and departments, and does not consistently include calls from home or other activities that do not directly result in billable CPT codes. Since most academic neonatologists are ≤0.8–0.9 cFTE and still generate >10,000 wRVUs per year, some benchmarks can be inflated (productivity/cFTE = 10,000/0.8 = 12,500 wRVUs/year). In contrast, salary benchmarks take total FTE and not the clinical component into consideration. Salary benchmarks are usually reported 6–18 months after data collection and are typically not adjusted for inflation. These factors may lead to a discrepancy between productivity and salary.

Surprisingly, there has been a recent upturn in neonatology wRVU benchmarks from 2024–25 to 2025–26. A similar upward trend was observed in combined academic and non-academic benchmarks after the COVID pandemic (Fig. 2). Standardizing data collection methodology and cFTE definitions across different benchmarks is needed to appropriately interpret and compare these data.

Administrators often use the productivity benchmarks to set thresholds for incentive compensation or bonuses. Behavioral economic principles suggest that linking compensation to higher productivity and patient volumes risks reduced physician time with patients and their parents resulting in an unsustainable “race to the bottom” [11, 12].

## “Resuscitation” of neonatal critical care physician workforce

To “resuscitate” the field, the following course corrections are proposed. These corrections are similar to the neonatal resuscitation algorithm where effective early intervention (such as initial stabilization and positive pressure ventilation—PPV) can prevent the need for advanced resuscitation (chest compressions and epinephrine) [13].

**Fig. 3:**
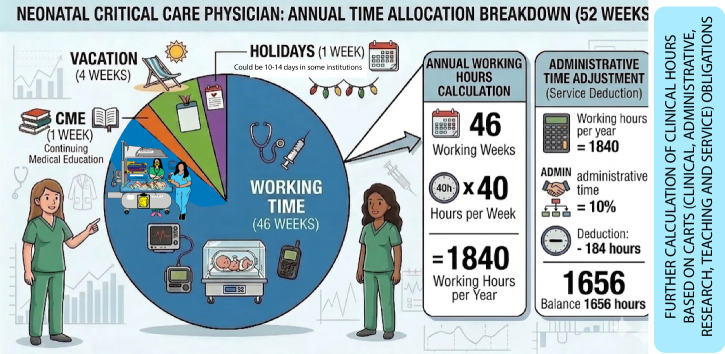
Annual work hour calculation in academic and non-academic settings in both inpatient and ambulatory pediatric care. CME continuing medical education. Created by Dr. Satyan Lakshminrusimha with Google Gemini.

1. **Initial Stabilization of staffing and work hours includes** prioritizing work-hour balance without salary reduction through better recognition of non-patient facing hours and administrative burdens. Offering flexibility in the work schedule and including hazard pay for night shifts are components of a recently developed staffing toolkit [14, 15]. We recommend a 40-h work week for 46 weeks per year (52 weeks minus 4 weeks for vacation, 1 week for holidays and one week for pursuing continuing medical education). This results in 1840 work hours per FTE (Fig. 3). Assuming 10% administrative and teaching time in both academic and non-academic settings, an optimal annual clinical load would be approximately 1656 hours. This tally is similar to ambulatory clinic work hours in both academic and non-academic settings. Similar to that environment, credit should be considered for calls from home at night and weekends, based on the intensity of phone calls and telemedicine requirements when off-site. For example, a night call that requires an average of 3–4 phone calls and the need to log in to the electronic medical record (EMR) to check X-rays and results could receive 20–25% credit (3–4 work hour credit for a 16-h night call) [6]. An approach often called the “one-minus” or CARTS approach (Fig. 4) determines clinical load (cFTE) by starting with 1.0 FTE and subtracting Administrative, Research, Teaching, and Service/Strategic planning time [16]. However, meticulous surveys conducted by the American Medical School Pediatric Department Chairs (AMSPDC) and Association of Administrators in Academic Pediatrics (AAAP) suggest that median clinical hours for academic neonatologists exceed 1900 h per year. The expected hours of work per year in the AAP/AMSPDC expectations survey were median (IQR) for Cardiac Intensive Care Unit (CICU): 1732 (1538, 2000), Hospital medicine: 1800 (1656, 1848), NICU: 1800 (1628, 1860), PICU: 1684 (1390, 1840) and pediatric emergency medicine: 1347 (1283, 1665) hours per year [3]. In one author’s institution (SL), work hour definitions were compared between three high-acuity divisions in pediatrics—pediatric emergency medicine (PEM), pediatric critical care, and neonatal critical care. Total work hours for 1.0 cFTE were 1316 h/y in PEM, 1792 h/y in pediatric critical care, and 1868 h/y in neonatal critical care. The difference in work hours per 1.0 cFTE between pediatric emergency medicine and pediatric and neonatal critical care are striking despite similar salaries [17]. In stark contrast, as mentioned previously, the expected three-year median wRVU benchmarks for these 3 divisions are 5337, 5300, and 10,664, respectively. Given the night calls, stress associated with high-risk deliveries, and resuscitation by neonatologists are similar to pediatric emergency medicine work, it is mind-boggling that neonatologists are expected to work 34% additional hours and generate twice the number of wRVUs compared to PEM. This “math isn’t mathing” and the difference in work hours and productivity between PEM and Neonatology defies logic.

**Fig. 4:**
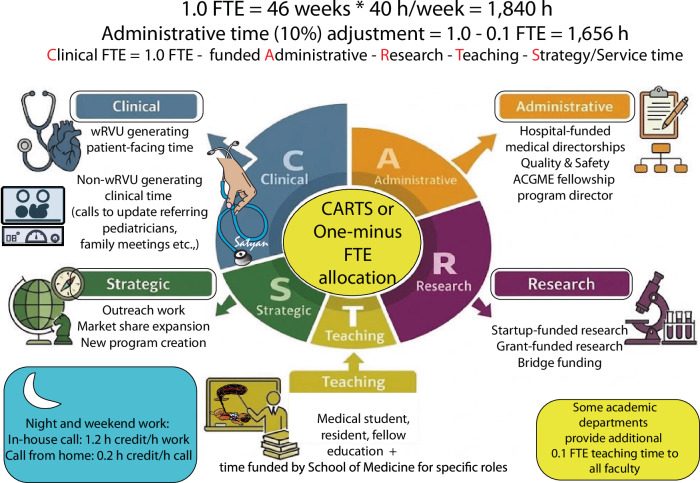
The one-minus or CARTS (clinical, administrative, research, teaching and service/strategy) model in academic medicine [16] adopted to neonatal critical care. The optimal work hours in academic and non-academic settings are shown. In most academic settings the clinical full-time equivalent (cFTE) work is adjusted for approximately 10% teaching effort. The “one-minus” approach refers to starting with 1.0 cFTE and subtracting time assigned for other activities to determine the precise cFTE hour per year. Created by Dr. Satyan Lakshminrusimha with Google Gemini.
2. **Gender equity**

The neonatal workforce is undergoing a significant demographic shift. By 2024, 73.6% of pediatric residents were female, and 75% of future neonatologists (current fellows) are female [18]. Consequently, gender equity is no longer just a moral imperative; it is a top priority for sustaining the workforce. Recent surveys have demonstrated gender inequity in compensation in neonatology [19].

Structural inequities remain, particularly regarding “pajama time” (after-hours documentation) and the inadequate support during childbearing and the first few years of parenthood. Proposals to address this include:

- **Correction of productivity metrics for childbearing leave:** While most neonatologists receive parental and childbearing leave with full pay, there are often no adjustments to productivity thresholds for earning a bonus. We recommend that productivity thresholds be proportional to actual clinical time and exclude child-bearing leave time, e.g., if the wRVU threshold for receiving a productivity-related incentive is 10,000 per year, a neonatologist who took 3 months childbearing leave should be eligible for this incentive at an adjusted threshold (10,000 × 9/12 months = 7500 wRVUs per year). We also recommend that bonuses based on profit-sharing be not reduced or prorated for leave ≤3 months in a fiscal year.
- **Lactation Holds and Credits:** Formally scheduling breaks in clinical workflows for milk expression, with lactation credits as needed to avoid financial penalties [20–22]. For outpatient clinics such as developmental follow-up clinics, we suggest providing a 25 min hold for every 4-h clinic session. To compensate for lost revenue from these sessions, 10% of outpatient wRVUs generated by that faculty member during the period of lactation (referred to as lactation value units or LVUs) are added to the total wRVUs. Similarly, on the inpatient side, 3% of wRVUs generated from the work in the NICU during the period of lactation are added to compensate for added “pajama” time to complete notes and other documentation as previously described. ^19^
- **Onsite Daycare:** Providing extended-hour facilities to align with neonatal shift work or flexible, as-needed childcare during periods of illness or specific weeks of daytime service only might benefit the neonatal workforce.

3. **The Identity Crisis: “Baby Docs” vs. Critical Care Specialists**
  Neonatology suffers from a public and institutional identity crisis. While a neurosurgeon is viewed with immediate prestige, neonatologists are often colloquially referred to as “baby docs”. This cultural perception extends to university structures, where small surgical specialties often have independent departments while neonatology remains a division within large pediatrics departments. These discrepancies exist despite the size of the neonatal workforce being larger than the many surgical specialist workforces such as urology and neurosurgery. Changing the name of our field from neonatology to neonatal critical care would recognize our work as having similar significance as our pediatric critical care and adult pulmonary critical care colleagues [23].
4. **Enhancing coding expertise and revision of wRVU credits to pediatric CPT codes**. Understanding the basics of neonatal coding is key knowledge for every practicing neonatologist. In addition, there is an urgent need for greater representation of pediatric subspecialists on the Relative value scale (RVS) Update Committee (RUC). The RUC is a 32-member multispecialty, volunteer committee from the American Medical Association (AMA). The composition of the RUC is partly based on specialties that account for high percentage of Medicare expenses resulting in one position dedicated to pediatrics. Specific lobbying to create modifiers for pediatric subspecialist care can increase the productivity of ambulatory pediatric specialists and reduce dependence on neonatology revenue for sustaining departments of pediatrics. In addition, benchmarking organizations should standardize the calculation of annual productivity. For both academic and non-academic settings, productivity should not be corrected for the standard administrative/teaching time given to all providers. For example, if an organization gives 0.1 FTE for administrative duties (Fig. 4) and productivity is 9000 wRVUs/y, these faculty members corrected productivity should be reported as 9000/1.0 cFTE and not as 9000/0.9 = 10,000/1.0 cFTE.
5. **Separate Departments:** If the above measures fail to improve engagement and morale in the neonatal critical care workforce, an evaluation of the feasibility of independent Departments of Neonatal Critical Care Medicine, potentially with separate residency tracks or a track with 2 years of pediatric residency and 3 years of fellowship should be considered [24]. This would allow for transparent “funds flow” models where other divisions are not financially dependent on neonatal revenue [25–27]. Such a proposal has recently received support from the European Society for Pediatric and Neonatal Intensive Care (ESPNIC) [28].

## Conclusion: a call for course correction

**Fig. 5:**
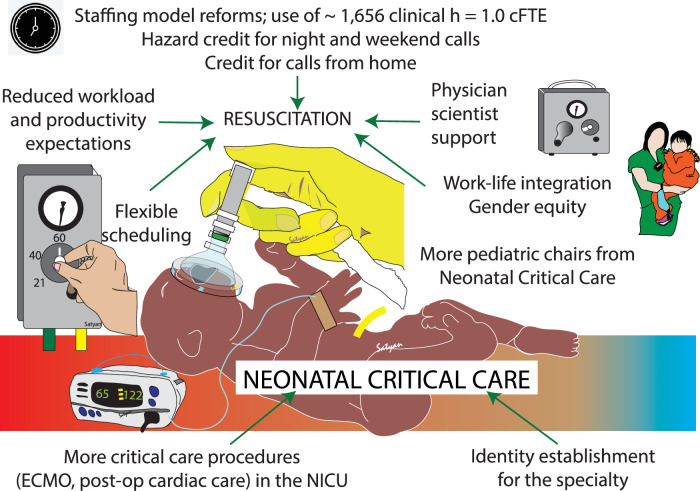
Resuscitation of neonatal critical care. Establishing standard staffing model reforms and steps necessary to improve engagement in the field of neonatal critical care. See text for details. Image copyright Satyan Lakshminrusimha.

The future of neonatal critical care depends on a fundamental shift in how the profession is valued and managed. Departments of pediatrics and chairs must move beyond the “tired horse” model of relying on neonatology to subsidize entire pediatric departments. By establishing clear professional identities, demanding gender-equitable workplace structures, aligning compensation with the true complexity of critical care, stabilizing staffing models and work hours, and having more neonatal critical care providers take on leadership roles to drive change in pediatric departments, we can ensure that the next generation of neonatologists find a sustainable and rewarding career (Fig. 5).
